# The Contribution and Therapeutic Potential of Epigenetic Modifications in Alzheimer’s Disease

**DOI:** 10.3389/fnins.2018.00649

**Published:** 2018-09-19

**Authors:** Ian C. Wood

**Affiliations:** School of Biomedical Sciences, Faculty of Biological Sciences, University of Leeds, Leeds, United Kingdom

**Keywords:** chromatin, epigenetics, Alzheimer’s, dementia, methylation, acetylation, ageing, histone

## Abstract

Alzheimer’s disease is a progressive neurodegenerative disorder, affecting 50 million people worldwide, for which there is no cure, or effective treatment. Individuals suffering from Alzheimer’s show a decline in cognition over time beginning with memory loss and ultimately leading to severe dementia, and inability to care for themselves. The cause of Alzheimer’s is not known but likely involves a combination of genetic, biochemical, and environmental factors. Some genes have been identified as risk factors but monozygotic twins discordant for Alzheimer’s disease suggest other factors must contribute to development of the disease. Investigation on epigenetic marks including DNA methylation and post-translational modifications of histones have shown that the patterns of these modifications change with age in the human population. Though individuals show specific differences in epigenetic marks at the individual gene level, there is a consistent pattern of epigenetic changes at the genome scale across the population. Similar changes have been identified in patients with Alzheimer’s disease, though these occur at an earlier age compared to healthy individuals. The early cognitive impairment in Alzheimer’s disease can be mistaken for premature ageing correlating with the timing of epigenetic changes occurring at a younger age in individuals with Alzheimer’s. Such observations suggest that the epigenetic changes may contribute to disease pathology. Exactly how epigenetic modifications contribute to specific aspects of Alzheimer’s disease is the focus of many researcher groups across the world. A number of drugs are available that inhibit the enzymes that modify chromatin and change the epigenetic landscape of the genome. Therefore, an understanding of the role of chromatin modifications in Alzheimer’s could offer an opportunity for novel therapeutic strategies. Research using animal models of Alzheimer’s suggests that the epigenetic changes in Alzheimer’s disease may have a profound impact on cognition and underlie cognitive impairment while there is no clear evidence that they might contribute directly to neuronal loss.

## Introduction

Alzheimer’s disease (AD) is a progressive neurodegenerative disorder and the most common form of dementia, affecting over 50 million people worldwide (Prince et al., 2015). The disease is characterised by memory loss, difficulties with communication and a loss of orientation, thought to be driven by neurodegeneration resulting from a build-up of extracellular amyloid plaques, and intracellular neurofibrillary tangles of hyperphosphorylated Tau protein. Individuals with AD show a progressive degeneration of cognitive function for which there is currently no cure or effective treatment and is ultimately fatal. AD is the third major cause of death in the developed world and it is predicted to cost the world economy $1trillion in 2018 (Prince et al., 2015). Although the cause of AD is not known, the most favoured hypothesis over the last 25 years has been the amyloid cascade hypothesis proposed in 1991, which suggested that inappropriate processing of the amyloid-β precursor leads to the build-up of amyloid plaques, formation of Tau tangles, and neuronal death (Hardy and Allsop, 1991). Though the amyloid hypothesis has provided biochemical targets for clinical intervention there is yet to be a therapeutically useful drug developed and, more recently, alternative hypotheses have been proposed [for a current overview see (Karran and De Strooper, 2016; Behl and Ziegler, 2017)]. Such hypotheses include; mitochondrial dysfunction, reduced glucose metabolism, cell cycle re-entry brought about by chemical stressors, reduced vasculature in the brain, and production of toxic oligomeric amyloid-β and the involvement of aberrant protein phosphorylation (Karran and De Strooper, 2016; Oliveira et al., 2017). There is not one single cause responsible for all cases of AD and AD can be divided into sporadic and familial cases. Familial AD is generally characterised by early onset (younger than 65 years) and associated with mutations in genes including; amyloid precursor protein (APP), presenilin (PSEN) 1, and PSEN2 (Bekris et al., 2010). Mutations in other genes such as Apolipoprotein E (APOE) and tau (MAPT) are associated with increased risk of sporadic, late onset AD though they not on their own sufficient to cause the disease (Coon et al., 2007; Allen et al., 2014). Monozygotic twins with discordance for AD suggest other factors, perhaps epigenetic mechanisms make significant contributions to disease pathology (Mastroeni et al., 2009).

Age is the biggest risk factor for AD and many epigenetic changes occur with normal ageing (Hernandez et al., 2011; Heyn et al., 2012). These changes are also seen in AD though they occur at an earlier age in Alzheimer’s patients (Wang et al., 2008; Chouliaras et al., 2013b) and AD can be mistaken for premature ageing (Burns and Iliffe, 2009). How these epigenetic changes contribute to the pathology and development of Alzheimer’s is currently of great interest.

The term epigenetics was coined by Conrad Waddington in 1942 (Waddington, 2012) in his description of development pathways of cells and the inheritance of acquired characteristics during cell division toward specialisation. Since that time, the phrase has been used in many different ways and many of the alternative definitions for it have been nicely summarised (Greally, 2018). One popular use of the word epigenetics, that will be used here, is for the description of modifications of DNA, such as cytosine methylation, and post-translational modifications of histones, such as acetylation, that provide long lasting changes to cells, and influence their homeostasis even in the absence of the original perturbation that generated them.

## DNA Methylation Changes Associated with Alzheimer’s Disease

The first identified, and most widely studied, epigenetic modification is DNA methylation. Cytosine residues on both strands, mostly within CpG dinucleotide sequences, can be methylated. This methylation contributes to gene regulation by acting as binding sites for proteins containing methyl cytosine binding domains that influence local chromatin structure. Once deposited, methylation patterns are maintained through cell division by DNA methyltransferase enzymes that methylate the hemi-methylated DNA in daughter cells. DNA methylation was, for a long time, considered to be irreversible and only subject to removal through the inhibition of re-deposition upon cell division. More recently a number of labs provided evidence that DNA methylation can be actively removed and that this removal plays a significant role in the function of the nervous system [reviewed in (Wu and Zhang, 2010)]. Compared to age-matched controls, individuals with Alzheimer’s show an overall global hypomethylation (Mastroeni et al., 2010; Chouliaras et al., 2013a) over the entire genome but hypermethylation of a number of specific genes (Wang et al., 2008). These differences reflect the changes associated with normal ageing (Fuke et al., 2004; Rakyan et al., 2010; Hernandez et al., 2011; Heyn et al., 2012) and is one piece of evidence supporting the idea that AD may, at least in part, be brought about by an accelerated ageing process. During the human lifespan, patterns of DNA methylation diverge over time between individuals, most convincingly demonstrated in monozygotic twins (Fraga et al., 2005; Talens et al., 2012). However, within older individuals (>75 years) patterns of DNA methylation appear to converge and undergo an age-dependent dedifferentiation demonstrated by a reduction in variation between tissues and specific brain regions, such as the cortex and cerebellum (Oh et al., 2016). This loss of variation in methylation is not the result of altered cell composition within tissues and appears more pronounced in individuals with Alzheimer’s (Oh et al., 2016).

Perturbation in normal DNA methylation can be brought about as a result of altered one-carbon methylation metabolism, resulting in altered levels of plasma homocysteine. Such altered one carbon methylation is associated with AD (Ho et al., 2011), although whether it contributes to pathology or is a marker is unclear (Seshadri, 2006; Troesch et al., 2016). The source of the methyl group added to DNA is the methyl donor, SAM, though SAM is also utilised in a number of other pathways and is a major cellular antioxidant. Excessive requirements in other pathways for example in response to oxidative stress has the potential to reduce the levels of SAM, and its availability for DNA methylation reactions. The global hypomethylation associated with Alzheimer’s is consistent with the lower levels of SAM seen in the brains of patients with Alzheimer’s (Morrison et al., 1996). Evidence that reduced one-carbon metabolism precedes changes in DNA methylation comes from animal models of Alzheimer’s fed a reduced vitamin B diet to reduce circulating levels of SAM. TgCRND8 mice (a mouse model of AD, **Table 1**^1^) on this diet show reduced DNA methyltransferase activity, hypomethylation of the *Psen1* promoter, increased PSEN1 expression, and increased level of amyloid plaques (Fuso et al., 2008; Fuso et al., 2011). Supplementing with SAM reduced expression of *Psen1* and *Bace1* in the TgCRND8 mice, and the development of amyloid plaques was decreased (Fuso et al., 2012; Cavallaro et al., 2017). Together, these data provide solid support for the idea that an imbalance in one-carbon metabolism results in reduced DNA methylation at the *Psen1* gene, increasing PSEN1 expression and activity, and contributes to the build-up of amyloid deposits. These data implicate the involvement of a single gene, although many other changes in DNA methylation are also likely to be present in these animals that may contribute to the increase in plaque load. Indeed, specific differences in DNA methylation have been observed between individuals with Alzheimer’s and age-matched healthy controls at number of genes associated with Alzheimer’s, including *PSEN1*, *APOE*, *MTHFR*, *DNMT1*, (Wang et al., 2008). The epigenetic drift that is observed during ageing may predispose individuals to the development of Alzheimer’s if it occurs more rapidly in some individuals or if specific genes are more susceptible to changes. Certainly epigenetic changes that influence expression of genes such as *DNMT1*, whose function is to regulate epigenetic marks, will likely result in a positive feedback loop, and an accelerated divergence from a normal ageing profile.

**Table 1:** Mouse models of Alzheimer’s disease.

Mouse model	Synonym	Genetic change	Link to AD	Consequence of change	Reference
Tg2576		APP KM670/671NL	The APP KM670/671NL mutation was found to be linked to AD in 2 large Swedish families	Results in an increase in Aβ40 and Aβ42 levels and mice develop amyloid pathology	Hsiao et al., 1996
APP/PS1	PS/APP	APP KM670/671NL, PSEN1 M146V	The PSEN1 M146V mutations has been found in a number of families and associated with AD	The PSEN1 M146V mutation results in reduced carboxypepdtidase γ-cleavage activity of PSEN1 and leads to increased levels of Aβ42	Holcomb et al., 1998
3xTg AD		APP KM670/671NL, MAPT P301L, and PSEN1 M146V	The MAPT P301L mutation has been found in 32 families around the world and is associated with fronto-temporal dementia and parkinsonism	The MAPT P301L mutation increases the aggregation rate of tau protein by promoting the formation of paired helical filaments	Oddo et al., 2003
CK-p25		Overexpression of p25 in postnatal forebrain	p25 stimulates CDK5 activity and aberrant CDK5 activity has been associated a number of neurodegenerative diseases. It is not clear if p25 expression is actually increased in individuals with AD	P25 is an activator of CDK5 and ectopic expression of p25 in neurons results in enhanced CDK5 activity	Cruz et al., 2003
APPswe/PSEN1dE9	APP/PS1	APP KM670/671NL, PSEN1dE9	The PSEN1dE9 mutation is found in AD patients that show large amyloid deposits and an absence of more typical dense amyloid deposits	The PSEN1de9 mutation results in increased amyloid deposition and an increase in the levels of Aβ42	Jankowsky et al., 2004
APPPS1-21		APP KM670/671NL and PSEN1 L166P	The PSEN1 L166P has been identified in a single family and associated with very early onset of AD	There is increased amyloid burden in mice at 6 months	Radde et al., 2006
TgCRND8		APP KM670/671NL, V717F	The V717F mutation has been found in a number of families and associated with early onset AD	Results in an increase in Aβ40 and Aβ42 levels and mice develop amyloid pathology.	Chishti et al., 2001


*The table identifies the different genetic changes used to develop mouse models of AD, how these mutations are linked to AD and what changes have been observed to occur in the mice as a consequence of the mutations.*

In addition to methylation, cytosines are also found hydroxymethylated (Kriaucionis and Heintz, 2009), though the level of hydroxymethylated cytosines are a small fraction of total cytosines (approx. 0.1–2%) and they were originally proposed to be an intermediate step toward DNA demethylation. Whether hydroxymethylated cytosines are an intermediate or act as a specific marker in their own right is still not certain. However, it is clear that their number increases in the brain during ageing and a gene ontology analysis uncovers an association of the genes showing hydroxymethylation with neurodegenerative disorders (Song et al., 2011). In a study of twins in which one twin suffered with AD while the other did not, lower levels of hydroxymethylated cytosine were found in both glia and neurones in the post-mortem brain of the AD sufferer (Chouliaras et al., 2013a). Consistent with other studies, the brain from the individual with AD also showed reduced levels of methyl cytosine. A second piece of evidence supporting the association of hydroymethylation and AD is the finding that a SNP within the gene encoding Ten-Eleven Translocation-1 (TET1, which catalyses the conversion of 5-methylcytosine to 5-hydroxymethylcytosine) is associated with late onset AD (Morgan et al., 2008). The SNP itself is in an exon but does not result in an altered protein coding sequence, and it is unknown whether the effect may impact positively, or negatively on either function, or expression.

Changes in DNA methylation may be important for the cognitive symptoms associated with Alzheimer’s rather than the neuronal loss. Aged mice show reduced levels of mRNA expression of the DNA methyltransferase DNMT3a2 which may underlie the reduced level of DNA methylation in older animals (Oliveira et al., 2012). Ectopic expression of DNMT3a2 in the brains of old mice using viral delivery increased their cognitive performance in fear conditioning and object-place recognition tasks. Additionally, reducing the level of DNMT3a2 using shRNA in younger mice reduced their performance across the same tests (Oliveira et al., 2012). Genetic association studies have identified SNPs in *DNMT3A* associated with mild cognitive impairment (Chouliaras et al., 2015), and aged mice show a reduction in the density of DNMT3 positive cells within the hippocampus as assessed by immunohistochemistry (Chouliaras et al., 2011). Where other *DNMT* family members have been interrogated for their association with Alzheimer’s, *DNMT1* did not show any association, while *DNMT3B* was found to be associated with Alzheimer’s in one study (Pezzi et al., 2014) though not in another (Coppede et al., 2012).

In summary the general picture emerging is that patterns of global DNA methylation show consistent changes with age and these changes appear to occur earlier in individuals with Alzheimer’s. To date there is little evidence that DNA methylation changes are responsible for the neuronal loss seen in AD, although given the fact changes in DNA methylation result in significant gene expression changes, it is not unreasonable to expect some impact on neuronal cell survival. There is however, substantial evidence that DNA methylation/demethylation plays a key role in cognition and experimental evidence demonstrating that altering the enzymes that modify methylation can have effects on learning and memory. Thus, it would seem that the DNA methylation changes associated with Alzheimer’s most likely contribute toward the cognitive deficits in individuals though this may be manifest in several ways for example altered expression of specific genes as well as a reduction in the ability to dynamically add and remove methylation. Therapeutically inhibiting these enzymes may provide some benefit and allow restoration of more normal levels of methylation. Increased DNA methylation is associated with many cancers and inhibitors of DNA methylation have been investigated as treatments for some cancers (reviewed in Nervi et al., 2015; Zahnow et al., 2016). A number of clinically tested DNA methylation inhibitors exist including Vidaza (5-azacytidine), Decabitine (5-aza-2′-deoxycytidine), Zebularine (1-(β-D-ribofuranosyl)-2(1H)-pyrimidinone) and Apresoline (Hydralazine), and there is some evidence to support efficacy of these drugs as cancer treatments. However, most trials have used these in combination with other anti-cancer therapies and some of these drugs are believed to work through their incorporation into newly synthesised DNA so while they may be effective in highly proliferative cancer cells they would be less useful in non-proliferative neurones. Toxicity appears favourable and even in combination with the histone deacetylase (HDAC) inhibitor, valproic acid, 5-azacytidine was well tolerated with no significant adverse effects in humans (Braiteh et al., 2008). The next step would be to characterise effectiveness of these inhibitors to improve cognition and monitor the longer term effects they may have within the nervous system.

## Histone Acetylation is Deregulated in Alzheimer’s Disease and Negatively Impacts Upon Memory

As well as modifications to DNA, histone proteins can be posttranslationally modified at specific residues leading to a change in the level of gene expression. This is brought about by an alteration of chromatin structure and/or recruitment of specific proteins to these epigenetic marks. One of the earliest modifications identified to occur on histones is the acetylation of lysine residues which is deposited by enzymes known as histone acetyltransferases (HAT) and removed by HDACs. Acetylation of histones is generally associated with active gene transcription. A number of studies have identified an important role for histone acetylation in normal ageing and in cognitive function. In aged mice, reduced memory performance has been seen to be associated with a reduction in histone acetylation levels (Peleg et al., 2010). In young mice, learning was associated with an increase of Histone H4 lysine 12 (H4K12) acetylation at genes whose expression was increased. By contrast, older (16 month) mice showed a lack of H4K12 acetylation and little change in gene expression. Treatment with the HDAC inhibitor SAHA was able to enhance fear conditioning induced gene expression changes and restore learning in aged mice, by promoting increases in acetylation. Aged mice also show an increased expression of HDAC2 within the hippocampus and this is thought to be associated with the normal ageing process as it can be attenuated by calorific restriction (Chouliaras et al., 2013b).

There is a wealth of evidence that links protein acetylation to AD and suggests that such changes in acetylation play an important role in underlying the cognitive deficits seen in patients. In brain sections from post-mortem samples of Alzheimer’s sufferers, both HDAC2 and HDAC6 show increased levels (Ding et al., 2008; Graff et al., 2012) though a separate report suggested reduced HDAC2, and HDAC1 levels (Mastroeni et al., 2010). The only study looking at histone acetylation levels in post-mortem brains suggested that these are higher in patients with Alzheimer’s than in age-matched controls (Lithner et al., 2013). A more consistent pattern has emerged in animal models of Alzheimer’s. Mouse models of Alzheimer’s show the same baseline of histone acetylation as wild type animals but a blunted increase in acetylation levels in response to fear conditioning (Francis et al., 2009). Several studies have also shown that HDAC inhibitors can reduce memory impairments in a number of different mouse models of Alzheimer’s. Sodium butyrate, sodium valproate and SAHA all restored contextual fear memory in APPswe/PS1de9 mice (**Table 1**), and increased the acetylation levels of histones in the hippocampus (Kilgore et al., 2010). A 6 week treatment of sodium butyrate to 14 month old APPPS1-21 mice (**Table 1**) resulted in increased histone acetylation levels in the hippocampus, increased expression of a number of genes involved with synaptic transmission and the mice also demonstrated improved associative memory (Govindarajan et al., 2011). Phenylbutrate treatment restored deficits in contextual fear conditioning for Tg2576 mice (**Table 1**) and this was associated with increased spine density in hippocampal neurones, reduced levels of amyloid-β and increased expression of genes involved in synaptic plasticity (Ricobaraza et al., 2011). The HDAC inhibitor, W2, improved performance in the Morris water maze, decreased amyloid-β levels, and also increased expression of the amyloid-β degrading enzyme MMP2 in 3xTg AD mice (Sung et al., 2013). Together these data suggest that HDAC inhibitors have potential utility to reduce cognitive deficits associated with Alzheimer’s. However, the lack of selective HDAC inhibitors and a comprehensive knowledge of their target proteins has precluded a thorough understanding of their mechanism of action.

The Alzheimer mouse model, CK-p25 (**Table 1**), in which p25 is over expressed in neurones, shows many hall marks of Alzheimer’s including neuronal loss, amyloid-β accumulation and memory deficits (Cruz et al., 2003). These mice show increased expression of *Hdac2* (but not *Hdac1* or *3*) (Graff et al., 2012). This increase in HDAC2 protein resulted in increased levels of HDAC2 at a number of genes important in synaptic function, a reduction in the level of histone H4 acetylation at their promoters, and reduced expression of their mRNAs. Knockdown of HDAC2 protein using shRNA was able to restore both histone acetylation and gene expression levels identifying it as a causative agent for these effects. The CK-p25 mice also show a reduction in the density of synapses in the hippocampus and behaviourally, a reduced performance in contextual fear conditioning, and the Morris water maze compared to control animals. Both the synaptic density and the performance in the memory tasks is improved once HDAC2 is knocked down, providing compelling evidence that HDAC2 levels are functionally important (Graff et al., 2012). Interestingly, knockdown of HDAC2 was not associated with an increase in neuronal number, suggesting that the increase in synaptic density and cognitive improvement do not require production of new neurones to replace those that were lost (Graff et al., 2012). Knockdown of HDAC2 was also shown to improve memory in APPswe/PSEN1dE9 mice and this was correlated with increased histone acetylation at the *Bdnf* gene and increased *Bdnf* mRNA (Hsiao et al., 2017). Many strategies aimed at treating Alzheimer’s are based around either prevention of neuronal loss or replacement of those neurones that have been lost. These data suggest that modulating the neurones that remain could provide a therapeutic benefit to individuals with Alzheimer’s. In humans, HDAC2 protein levels are found at higher levels in post-mortem brain tissue from patients who suffered with Alzheimer’s compared to age-matched controls suggesting that targeting HDAC2 may be therapeutically useful (Graff et al., 2012).

Alongside the evidence that increased HDAC2 levels contribute to Alzheimer’s there is also substantial evidence that modulating HDAC2 levels has a direct effect on cognitive ability (Guan et al., 2009; Morris et al., 2013). Transgenic mice with increased expression of HDAC2, but not HDAC1, in neurones show a reduced performance in conditioned fear response and the Morris water maze (Guan et al., 2009). HDAC2 overexpression does not result in any obvious brain morphology deficiencies by 6 months, suggesting that increased HDAC2 alone does not lead to AD (Guan et al., 2009). The loss in cognitive performance resulting from increased HDAC2 correlated with a reduced density of dendritic spines of hippocampal CA1 pyramidal neurones and reduced LTP within the hippocampus. Interestingly, mice lacking HDAC2 showed a higher density of spines from equivalent neurones, enhanced performance in the memory tasks, and increased synaptic plasticity demonstrated by more easily induced LTP (Guan et al., 2009). Lack of HDAC2 in adult forebrain neurones is associated with an improved performance in associative memory tasks and enhanced LTP in hippocampal neurones (Morris et al., 2013). Together these data suggest that inhibition of HDAC2 in patients with Alzheimer’s may be able to compensate for some of the cognitive deficits, but would probably have little effect on the neurodegeneration associated with Alzheimer’s. Nevertheless, providing cognitive improvements to individuals would provide significant enhancements to their quality of life. The mechanism by which HDAC2 negatively regulates synaptic plasticity and memory is unknown but might involve its interaction with the transcription factor Sp3 (Yamakawa et al., 2017). Like HDAC2, Sp3 is elevated in the hippocampus of post-mortem brains from Alzheimer’s patients, and is also upregulated in the CK-p25 AD mouse model (Yamakawa et al., 2017). Knockdown of Sp3 using shRNA in CK-p25 mice was sufficient to increase excitatory postsynaptic currents in neurones, restore expression levels of repressed genes, and enhance LTP. Evidence that a HDAC2:Sp3 complex is important for these features was provided by the observation that expression of a C-terminal region of HDAC2, that interacts with Sp3 and thus disrupt the endogenous HDAC2:Sp3 complex, was able to mimic the effects seen with either Sp3, or HDAC2 knockdown (Yamakawa et al., 2017). Use of this C-terminus of HDAC2 as a therapeutic agent would be quite challenging given its large size, though its effectiveness in animal models should provide enough preliminary evidence to encourage the development of a small molecule that could interfere with the HDAC2:Sp3 interaction and equally provide potential benefit to human patients.

Evidence that the increased levels of another HADC family member, HDAC6, seen in patients with Alzheimer’s contributes to disease pathology was highlighted by Govindarajan et al. (2013). Knockout of *Hdac6* in the APPPS1-21 mouse (**Table 1**) rescued impaired freezing behaviour and improved performance in the Morris water maze suggesting that removal of *Hdac6* was sufficient to restore associative and spatial memory. Unlike HDAC2, HDAC6 is found in the cytoplasm and its most recognised target protein is tubulin (Zhang et al., 2003), though it can also deacetylate Tau and Prx1 (Cook et al., 2014; Carlomagno et al., 2017). Deacetylation of Tau increases its potential to be phosphorylated and potentially contributes to aggregation (Carlomagno et al., 2017) while deacetylation of Prx1 reduces it anti-oxidant activities (Choi et al., 2017). A number of independent studies highlight increased HDAC6 expression associated with Alzheimer’s and inhibition of HDAC6 as potentially beneficial, though the evidence would suggest that the important proteins regulated by HDAC6 are not localised to the nucleus and therefore not directly involved in regulating gene expression. Additionally many of the HDAC inhibitors that have been shown to improve cognition in Alzheimer’s mouse models do not effectively inhibit HDAC6, indicating that they must be providing their beneficial effects via an alternative HDAC, most likely HDAC2.

Levels of protein acetylation are regulated not just by removal via deacetylases but also by addition via acetylases. In complimentary studies to those identifying increased HDAC levels, reduced levels of the histone acetyltransferase CREB-binding protein (CBP) were observed in APPswe/PSEN1dE9 mice (**Table 1**) at an age of 3 months, preceding the development of Alzheimer’s like symptoms, and this was associated with reduced histone acetylation levels in the hippocampus (Ettcheto et al., 2017). Additionally ectopic expression of CBP in 3xTg AD mice using lentiviral delivery enhanced performance of these mice in the Morris water maze (Caccamo et al., 2010). Thus it would appear that shifting of the acetylation equilibrium toward the non-acetylated state has a negative impact on cognitive function, though there is no suggestion that it plays any significant role in cell survival and most likely does not directly contribute to neuronal loss in AD.

Global inhibition of HDAC activity in the brain is unlikely to provide long term benefits, as activity of some deacetylase enzymes have been positively correlated with neuronal survival and/or cognition. Evidence has implicated a role for HDAC1 in facilitating DNA damage repair (Kim et al., 2008; Dobbin et al., 2013; Wang et al., 2013). In CK-p25 mice, p25 can interact with HDAC1 and inhibit its recruitment to chromatin and its repressor activity (Kim et al., 2008). Specific knockdown of HDAC1 or inhibition of HDACs with MS-275 led to DNA damage and neuronal death of primary cortical neurones while ectopic expression of an enzymatically active form of HDAC1, but not HDAC2, was able to rescue p25 induced cell death (Kim et al., 2008). HDAC1 facilitates DNA repair via its interaction with an NAD-dependant deacetylase, SIRT1 (Dobbin et al., 2013). HDAC1 itself is acetylated, on a lysine residue at amino acid position 432, which causes a reduction in HDAC1 activity, and this acetyl mark is removed by SIRT1. Treatment of CK-p25 mice with activators of SIRT1 results in deacetylation of HDAC1 and a reduction in DNA damage as assessed by lower numbers of γH2AX positive cells (Dobbin et al., 2013). HDAC1 and HDAC2 are structurally very similar and there are currently no inhibitors selective for one over the other. Thus any potential benefits in using a HDAC inhibitor to inhibit HDAC2 may be counter balanced by the detrimental effects of the inhibition of HDAC1.

Unlike Class I HDACs (1, 2, 3, and 8), which are expressed in the nucleus, class II HDACs shuttle between the nucleus and cytoplasm. In neurones, HDAC5 functions in the nucleus to repress gene expression, and is inactivated by exportation from the nucleus in response to activation of a calcium signalling pathway driven by neuronal activity (Chawla et al., 2003). Knockout of HDAC5 in the APPPS1-21 mouse (**Table 1**) model did not produce an improvement in memory in either fear conditioning or performance in the Morris water maze (Agis-Balboa et al., 2013). The result of HDAC5 knockout in wild type mice is not clear as one group identified reduced performance in a Morris water maze by *Hdac5* knockout mice (Agis-Balboa et al., 2013) while another group reported that *Hdac5* knockout had no effect on context dependent fear memory (Kim et al., 2012). Knockout of another class II HDAC, *Hdac4*, specifically in forebrain neurones resulted in reduced synaptic plasticity, and impaired performance in context dependent memory tasks (Kim et al., 2012). Haploinsufficiency of HDAC4 is also associated with mental retardation in humans (Williams et al., 2010) suggesting that the roles identified for HDAC4 in mice are likely conserved in humans.

However, increasing histone acetylation levels globally may also have some negative consequences with respect to proteins that recognise and interact with acetylated lysine. Acetylated histones provide binding sites for specific proteins that contain a binding motif known as a bromodomain (Dhalluin et al., 1999). One set of proteins known to bind chromatin via an interaction of such a domain with acetylated histones are the BET proteins (Padmanabhan et al., 2016). JQ1 is a small molecule able to block the binding of these proteins to chromatin, and can improve associative memory in the APPS1-21 transgenic mice, as well as enhance LTP of hippocampal neurones (Benito et al., 2017). Treatment of mice with JQ1, globally or specifically in the brain, immediately following a fear conditioning protocol resulted in increased freezing behaviour at 24 h and 4 weeks after training, and enhanced performance in the Morris water maze. Associated with the improved memory was an increase in LTP of hippocampal slices treated with JQ1 (Benito et al., 2017). Interestingly JQ1 was able to restore memory and LTP levels in the APPS1-21 mice to those seen in wild type control animals, and was also able to enhance both memory and LTP when tested in wild type mice (Benito et al., 2017). Another study, however, showed a lack of effect on response to a fear conditioning stimulus in wild type mice receiving a 3 week treatment of JQ1 and showed evidence that memory consolidation was negatively affected (Korb et al., 2015). Such apparent differences may be the result of different conditioning paradigms. As we already know that memory formation and recall is a complex process, such apparently conflicting data in the literature is not surprising. Perhaps more focus on the AD model is warranted rather than examination of the effects on wild type animals. Treatment of APPS1-21 mice with JQ1 was also shown to change the expression of a number of genes important for synaptic function, although it is unclear which of these are important and whether these changes are the result of inhibition of BET protein interaction with acetylated histones or other acetylated proteins. By contrast wild type animals showed fewer changes in gene expression in response to JQ1. This pattern of response is mimicked by treatment with HDAC inhibitors and has led to the suggestion that the chromatin state in Alzheimer’s becomes deregulated and thus much more amenable to the influence of external factors (Fischer, 2014).

Inhibitors of both BET and HDAC are of interest, like DNA methylation inhibitors, for their potential as anti-cancer therapies. There has been no evidence so far that HDAC, or BET inhibitors can reduce, or stop the ongoing neurodegeneration in Alzheimer’s, though their effectiveness at improving cognitive ability after the onset of disease pathology does show promise for reducing some of the more tragic consequences of AD. Targeting acetylation globally with HDAC inhibitors is likely to have significant deleterious effects due to the positive roles of some HDAC enzymes in DNA damage repair, as well as learning, and memory. Despite an often cited need for more selective or specific HDAC inhibitors, none have yet emerged. The similarity of the structures of the active site within HDAC enzymes would suggest that traditional small molecule type drugs would not be able to interact one active site but be excluded from another. Differences in function between the HDAC family members are brought about by their interaction with different proteins and an approach aimed at inhibiting these interactions might prove more rewarding and more amenable to produce selective inhibitors. However, inhibiting protein:protein interactions is challenging and brings a different set of hurdles to overcome before success can be made. A combination of HDAC and DNA methylation inhibition has been trialed in cancer (Braiteh et al., 2008) and may be warranted as a potential treatment of AD. The major advantage of HDAC inhibition over inhibition of DNA methylation is that we know HDAC inhibitors can increase cognitive ability in mouse models of AD over the short term. The barrier to overcome with HDAC inhibition is likely to be the lack of compounds that can selectively inhibit one member of the HDAC family without interfering with the others.

## Increased Histone Methylation Negatively Impacts on Cognitive Function in Alzheimer’s

The other major post-translational modification that is present on histones is methylation. Both lysine and arginine amino acids can be methylated and several forms of methylated residues exist including mono-, di- or tri- methylated lysines and mono-, symmetrically di-, and asymmetrically di-methylated arginines (Di Lorenzo and Bedford, 2011). Unlike acetylation, methylation can have positive or negative effects on transcription depending on which residue is methylated. For example methylation of Histone H3 lysine 4 (H3K4) is associated with gene expression (Santos-Rosa et al., 2002) while methylation of H3K9 and H3K27 is associated with gene repression (Rea et al., 2000; Kuzmichev et al., 2002). Very few studies have so far investigated a potential role for histone methylation in the pathology of AD and most of what we can surmise about their role in Alzheimer’s pathology has been determined using cultured neurones or other cells. Some analysis has been performed in mouse models and it is suggested that Tg2576 mice (**Table 1**) show increased levels of dimethylated H3K9 (H3K9Me2) in both the cortex and hippocampus by 3 months of age (Lithner et al., 2013). However, in the same study, they also appear to show an increase in acetylation of H3 and reduced levels of total histone protein in relation to the total cellular protein, which may skew the apparent differences as these were used for normalisation (Lithner et al., 2013). Exposure of cortical and hippocampal neurones to hypoxia resulted in an increase in H3K9Me2 and decrease in H3 acetylation in the Neprilysin promoter (Wang et al., 2011). Neprilysin is the enzyme that degrades amyloid-β peptide, regulating its levels, and reduced Neprilysn is associated with increased amyloid-β (Marr et al., 2004). The increased H3K9Me2 levels are mostly likely brought about as a result of increased levels of the major histone methyltransferse which deposits this mark, G9a. Knockdown of G9a using siRNA caused a reduction in H3K9Me2 though was not sufficient on its own to restore NEP gene expression (Wang et al., 2011). If increased activity of G9a has a contributory role in AD then G9a inhibitors could have some utility in its treatment. Drugs that inhibit G9a have been identified and one, diazepin-quinazolin-amine derivative (BIX-01294) shows specificity for G9a over other methyltransferases (Kubicek et al., 2007), is able to modulate H3K9Me2 levels in mammalian cells, induces autophagy in a number of tumour cell lines (Ciechomska et al., 2016), and reduces tumour growth in a mouse model (Ke et al., 2014). Perhaps most encouragingly, treatment with BIX-0294 was able to restore synaptic plasticity that had been impaired in rat hippocampal slices by amyloid-β 1-42 oligomers (Sharma et al., 2017). Thus G9a inhibition may be one way to counter synaptic plasticity deficits thought to be associated with AD and thus improve patient cognition.

Histone methylation also occurs at H3K4, in this case associated with increased gene expression. Induction of p25 expression in the CK-p25 mouse results in increased tri-methylated H3K4 (H3K4Me3) at 3,667 promoter regions and decreased H3K4Me3 at 5,056 promoter regions, with corresponding increased and decreased expression of the associated genes (Gjoneska et al., 2015). This data was collected from homogenised hippocampal tissue and not all cells may respond in the same way, for example increased H3K4Me3 present at some genes may occur in different cells to the decreased H3K4Me3 present at others. Indeed, the promoters showing increased H3K4Me3 are linked to genes associated with immune function, suggesting a microglial origin while the promoters showing decreased H3K4Me3 are linked to genes associated with neurodevelopment suggesting a neuronal origin. Consistent with this idea, forebrain specific loss of the methyltransferase enzyme KMT2A, which adds H3K4Me3 to histone proteins, results in reduced expression of a similar set of genes to that seen in CK-p25 mice (Kerimoglu et al., 2017). Loss of KMT2A in forebrain neurones also resulted in reduced fear conditioning and impaired spatial memory in the Morris water maze (Kerimoglu et al., 2017). The gene targets of the enzyme included those involved with transcriptional regulation, chromatin binding and mRNA processing, reduction in expression of which is likely to have broad effects on cellular physiology. Overall, the behavioural phenotype of mice lacking KMT2a is very similar to that of the CK-p25 mice used as a model of Alzheimer’s, though the pathology is different as KMT2A knockdown does not result in the associated neurodegeneration. These data do provide some suggestions that a reduction in H3K4Me3 in neurones contributes to the cognitive decline in Alzheimer’s, while increased H3K4Me3 in microglia or other immune cells may contribute to the neuro-inflammatory response. Therapeutically, enhancing KMT2a activity would be challenging, although inhibiting the opposing enzymatic activity, H3K4Me demethylase, would be tractable, and could achieve the same objective of increased H3K4Me in neurones. A number of demethylases have been identified that target H3K4Me3 including KDM2B (Fbxl10, Janzer et al., 2012) and the KDM5A-D family (JARID1-4, Secombe and Eisenman, 2007) and selective inhibitors to them have been identified (Rotili and Mattevi, 2016).

## Conclusion and Future Perspective

In summary, individuals with AD display a host of epigenetic differences compared to healthy age-matched individuals. These chromatin changes are also associated with normal ageing and give support to the idea that AD is at least partly a manifestation of a faster ageing process. The changes in chromatin marks associated with Alzheimer’s appear to have a demonstrable negative impact on cognitive function, but any direct contribution to neurodegeneration itself has not been well established. Treatments or other strategies that target the enzymes regulating these chromatin marks have been shown to have beneficial effects on cognition in mouse models of Alzheimer’s, without altering neuronal survival. Previous strategies for the treatment of Alzheimer’s have focused on the clearance of amyloid-β deposits, reduction of the hyperphosphorylated Tau, and inhibition of neuronal death and/or replacement of lost neurones. However, even after some success in animal models, none of these approaches have proven to be a successful strategy for treating AD in humans. Approaches to influence the deposition or removal of specific chromatin marks represent so far untried strategies, which might be expected to provide cognitive benefits and at least improve the quality of life for Alzheimer’s sufferers. However, with continued neuronal loss, such treatments would be expected to lose efficacy over time, so perhaps a dual strategy targeting chromatin changes coupled with methods to enhance neuronal survival would be required for any long term effective therapy.

## Author Contributions

IW wrote the manuscript.

## Conflict of Interest Statement

The author declares that the research was conducted in the absence of any commercial or financial relationships that could be construed as a potential conflict of interest.

## Footnotes

1 A number of mouse models have been developed to provide an *in vivo* model of Alzheimer’s disease that replicates the condition seen in humans. None of the models replicates all features of Alzheimer’s seen in humans and many drugs that show efficacy within these models have been found to be less efficacious in human patients. A list of the models discussed in this review, the genetic basis of the model, and their origin are given in **Table 1**.

## Abbreviations

AD: Alzheimer’s disease
BET: bromodomain and extra-terminal
HDAC: histone deacetylase
SAM: S-adenosylmethionine
SAHA: suberanilohydroxamic acid
